# Spontaneous Cure of Ovarian Tumor

**Published:** 1876-08

**Authors:** Owen Wright


### Spontaneous Cure of an Ovarian Tumor.

By OWEN WRIGHT, A. M., M. D.

On the 20th of November I was called to see Mrs. A. B. I arrived at the house at 4 o'clock, P. M. She was expecting to be confined at any hour, but was not presenting any symptoms of labor. At 10 o'clock of the same day she was taken with a chill and fever; and when I saw her the breathing was 28, the pulse 200 per minute. I was not able to ascertain the cause of the excitement, although I knew it was of local origin. I remained with her all night, and met the indications for treatment according to my abilities. At 5 o'clock the next morning, labor came on and terminated favorably in one hour. It was not until after the child was born that I was able to discover the cause of the chill and fever.

In the region of the left ovary was a tumor, that the lady had never noticed; she was so very large (although a medium-sized woman) that I did not find it, although I passed my hand over that region several times. The patient was so feeble, that I gave her constitutional treatment the following four weeks. All this time the tumor continued to enlarge; and its pressure from within outwards, caused an injury to the external parts, immediately above the os pubis.

My diagnosis was a cystic tumor of the ovary; and the 30th day after delivery, I performed the operation of paracentesis, and drew off about one gallon of serous fluid. It continued to discharge one week, during which time the parts exterior, all of three by five inches, sloughed away, so that the remains of the tumor could be seen. During another week the wound failed to heal, and a second like tumor reached half the size of the former. At this period the prospects of success were not very flattering. Another week under treatment and the tumor burst, and almost at the same time the fluid entered the womb through the fallobian tube and was discharged through the vagina. After this the wound began to heal: the vaginal discharge continued occasionally another week. The constitutional symptoms improved rapidly, and at the end of three months the mother walked out doors.

During her pregnancy the mammary glands did not increase in size. I do not think either of then would have weighed over one ounce, till about the ninth week after her delivery, when she noticed that her breasts were filling.

She is about 30 years old; her weight is 130 pounds. She has borne two other children, and each time was afflicted with nursing sore mouth.—*Chicago Medical Journal and Examiner.* [May this not have been tubal dropsy or a cyst of the broad ligament, Ed.]

